# Supplementary material for: Optical Properties and Light-Induced Charge Transfer in Selected Aromatic C60 Fullerene Derivatives and in Their Bulk Heterojunctions with Poly(3-Hexylthiophene)
Source: Materials (Basel). 2022 Oct 5;15(19):6908. doi: 10.3390/ma15196908 (PMC9571621; doi:10.3390/ma15196908)
Supplement: Supplementary file 1 [file materials-15-06908-s001.zip › materials-1919840-supplementary.pdf]

materials

MDPI

Article

Optical Properties and Light-Induced Charge Transfer in Selected Aromatic C60 Fullerene Derivatives and in Their Bulk Heterojunctions with Poly(3-Hexylthiophene)

Maciej Krajewski [1,*], Piotr Piotrowski [2], Wojciech Mech [1], Krzysztof P. Korona [1], Jacek Wojtkiewicz [1], Marek Pilch [1], Andrzej Kaim [2], Aneta Drabińska [1] and Maria Kamińska [1]

1 Faculty of Physics, University of Warsaw, Pasteura 5, 02-093 Warsaw, Poland; wojciech.mech@fuw.edu.pl (W.M.); krzysztof.korona@fuw.edu.pl (K.P.K.); jacek.wojtkiewicz@fuw.edu.pl (J.W.); marek.pilch@fuw.edu.pl (M.P.); aneta.drabinska@fuw.edu.pl (A.D.); maria.kaminska@fuw.edu.pl (M.K.)
2 Faculty of Chemistry, University of Warsaw, Pasteura 1, 02-093 Warsaw, Poland; ppiotrowski@chem.uw.edu.pl (P.P.); akaim@chem.uw.edu.pl (A.K.)
* Correspondence: maciej.krajewski@fuw.edu.pl

Supplementary Materials

1. Synthesis of new fullerene derivatives

Details regarding synthesis and characterization of the obtained fullerene derivatives containing pyrene functional group ([C60]P1 and [C60]P2) are described in our recent paper [21]

Herein, we describe the synthesis procedure of fullerene with tiophene functional groups ([C60]T1 and [C60]T2). Malonate substrates were synthesized according to the method described by De la Torre, et al. in Synthesis of [60]-fullerene-quercetin dyads (Tetrahedron Letters 2002, 43, 4617–4620, https://doi.org/10.1016/S0040-4039(02)00867-5).

Ethyl(2-(thiophen-3-yl)ethyl) malonate

Solution of ethyl malonyl chloride (301 mg, 2 mmol) in anhydrous dichloromethane (5 ml) was added dropwise for 20 min to a well stirred solution of 3-thiopheneethanol (256 mg, 2 mmol) and triethylamine (202 mg, 2 mmol) in dry methylene chloride (20 ml) at 0°C under argon atmosphere. The resulting mixture was then heated to room temperature and stirred for an additional 16h. After evaporation of the solvent, using a rotary evaporator, the resulting crude product was chromatographed on silica gel using *n*-hexane/ ethyl acetate (4:1,v/v) as eluent. The desired product was obtained as a colorless oil.

Yield: 91%, ESI-MS: 265.1 $[M+Na]^+$, FT-IR (neat) $\nu_{max}(cm^{-1})$ 2983.31, 1748.64, 1728.72, 1463.20, 1412.31, 1368.06, 1330.44, 1268.48, 1186.62, 1146.79, 1029.52, 943.22, 861.35, 834.80, 777.27, 686.55, 662.21, 633.45, 602.79; ^{1}H NMR (300 MHz, $CDCl_3$) δ 7.27 – 6.95 (m, 3H), 4.35 (t, J = 6.9 Hz, 2H), 4.18 (q, J = 7.1 Hz, 2H), 3.36 (s, 2H), 2.99 (t, J = 6.9 Hz, 2H), 1.26 (t, J = 7.1 Hz, 3H) ppm; ^{13}C NMR (125 MHz, $CDCl_3$) δ 166.51, 166.35, 128.12, 125.65, 121.60, 65.25, 61.47, 41.52, 29.19, 14.00ppm.

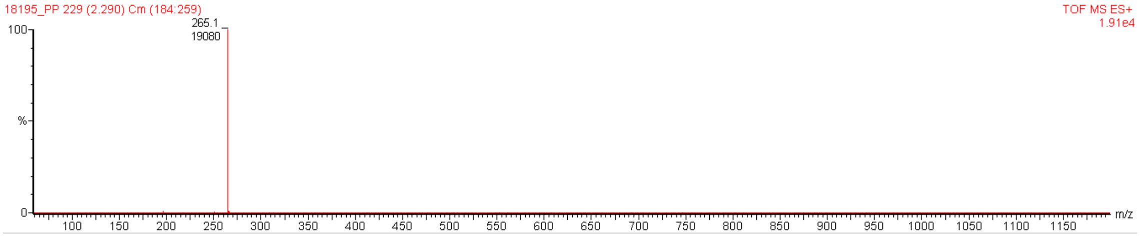

Figure S1. ESI-MS spectrum of ethyl(2-(thiophen-3-yl)ethyl) malonate as a [M+Na]$^+$ cation.

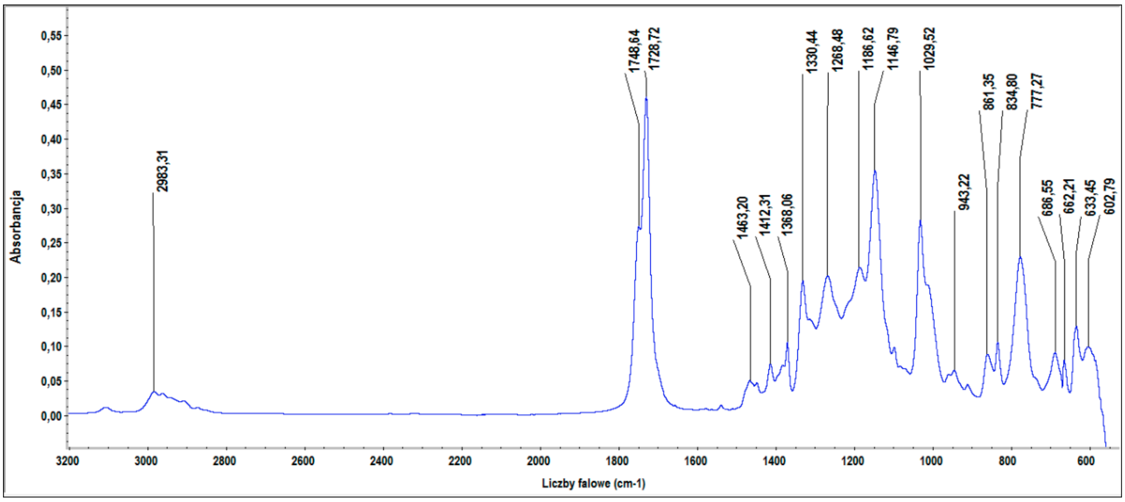

Figure S2. IR spectrum of ethyl(2-(thiophen-3-yl)ethyl) malonate (neat).

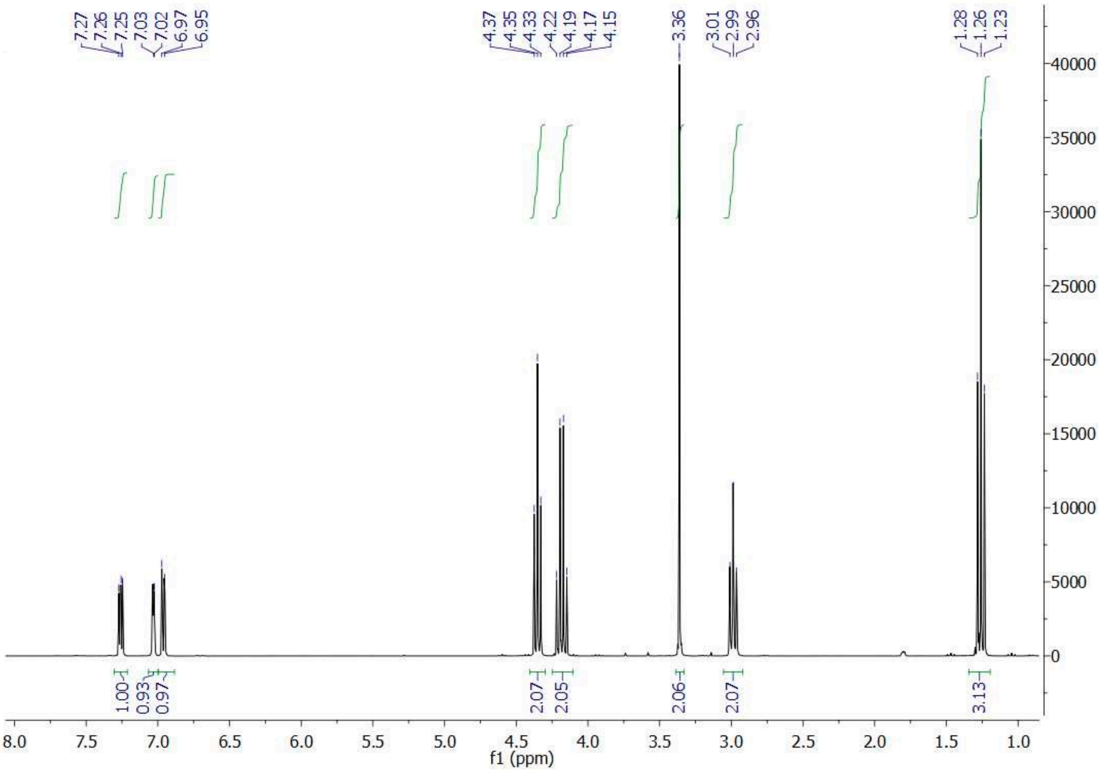

Figure S3. ^{1}H NMR spectrum of ethyl(2-(thiophen-3-yl)ethyl) malonate in $CDCl_3$.

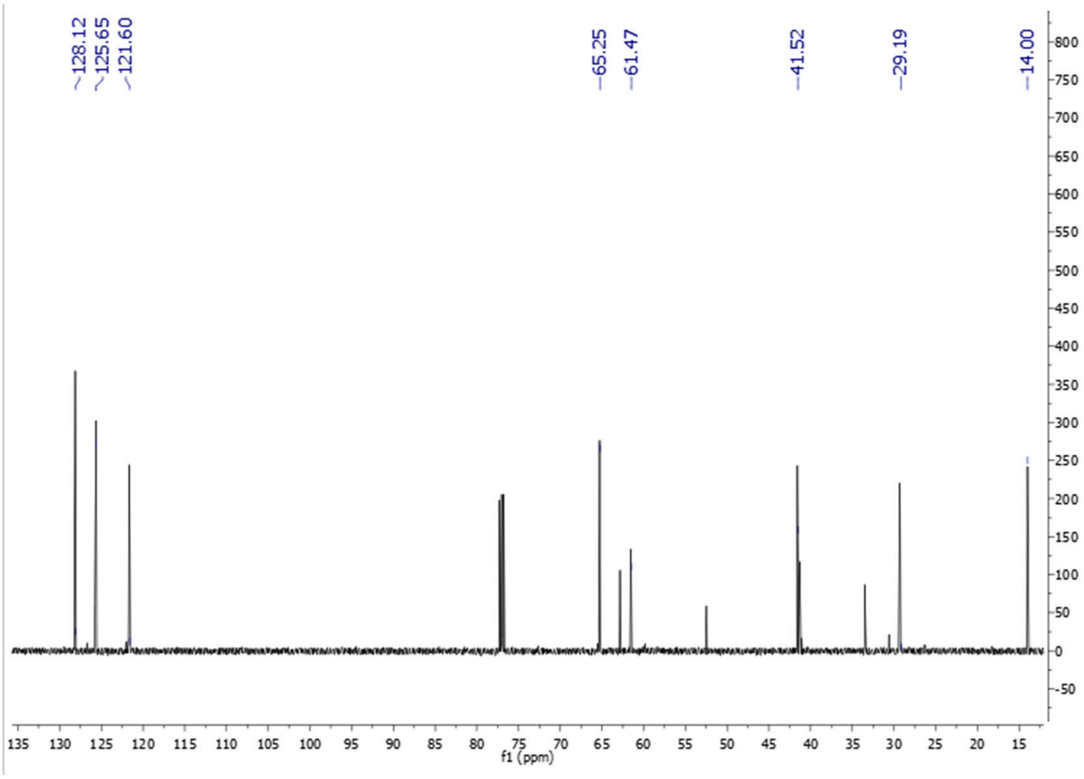

Figure S4. ^{13}C NMR spectrum of ethyl(2-(thiophen-3-yl)ethyl) malonate in $CDCl_3$.

Fullerene derivatives[C60]T1 and [C60]T2 were synthesized according to modified Bingel method.[18, 19]

61. -Etyloxycarbonyl-[61-(2-(thiophen-3-ylo)ethylo)]-1,2-methano[60]fullerene ([C60]T1)

To a solution of C_{60} (144 mg, 0.2 mmol) in anhydrous toluene (100 ml) a solution of ethyl(2-(thiophen-3-yl)ethyl) malonate (24 mg, 0.1 mmol) in toluene (5 ml), a solution of iodine (25 mg) in toluene (10 ml) and DBU (31 µl, 0.2 mmol) were added. The obtained mixture was vigorously stirred for 18h at room temperature under a arggon atmosphere. After concentration under reduced pressure the obtained mixture was chromatographed using toluene/n-hexane (1:2, v/v) as eluent, to yield **[C60]T1** as a brown powder.

Yield: 46%, ESI-MS: 983.4 $[M+Na]^+$), FT-IR (KBr) $\nu_{max}(cm^{-1})$ 2966.92, 1742.49, 1651.62, 1460.80, 1426.73, 1294.97, 1267.71, 1231.37, 1201.83, 1185.93, 1095.07, 1015.56, 995.11, 861.08, 833.32, 767.94, 702.07, 636.19, 579.40, 549.86, 527.15 ; ^{1}H NMR (300 MHz, $CDCl_3$) δ 7.31-7.06 (m, 3H), 4.73 (t, *J* = 6.7 Hz, 2H), 4.51 (q, *J* = 7.1 Hz, 2H), 3.20 (t, *J* = 6.7 Hz, 2H), 1.44 (t, *J* = 7.1 Hz, 3H) ppm; ^{13}C NMR (125 MHz, $CDCl_3$) δ 163.55, 163.41, 145.28, 145.22, 145.17, 145.14, 145.11, 145.07, 144.84, 144.64, 144.57, 144.45, 143.83, 143.03, 142.97, 142.95, 142.16, 141.90, 141.88, 141.86, 140.91, 139.00, 138.90, 137.21, 129.39, 128.09, 125.92, 121.86, 66.80, 63.34, 54.08, 29.39, 14.18ppm.

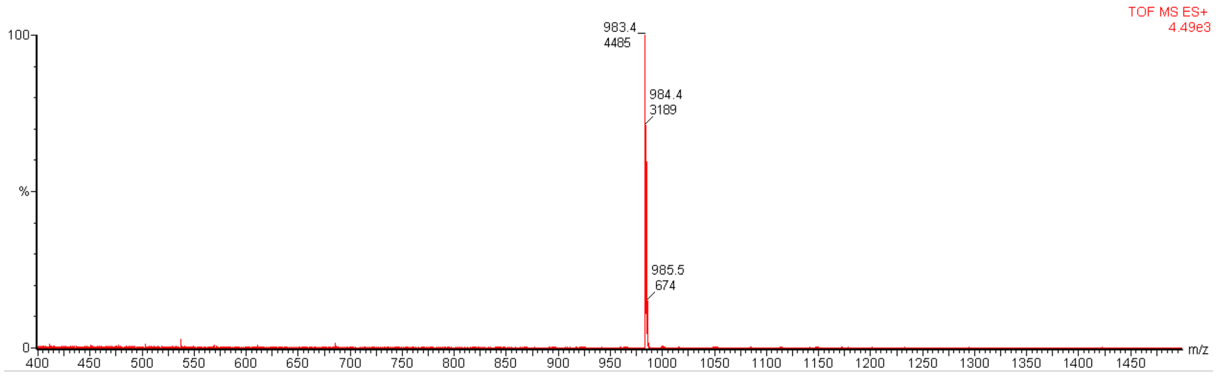

Figure S5. ESI-MS spectrum of **[C60]T1** as $[M+Na]^+$ anion.

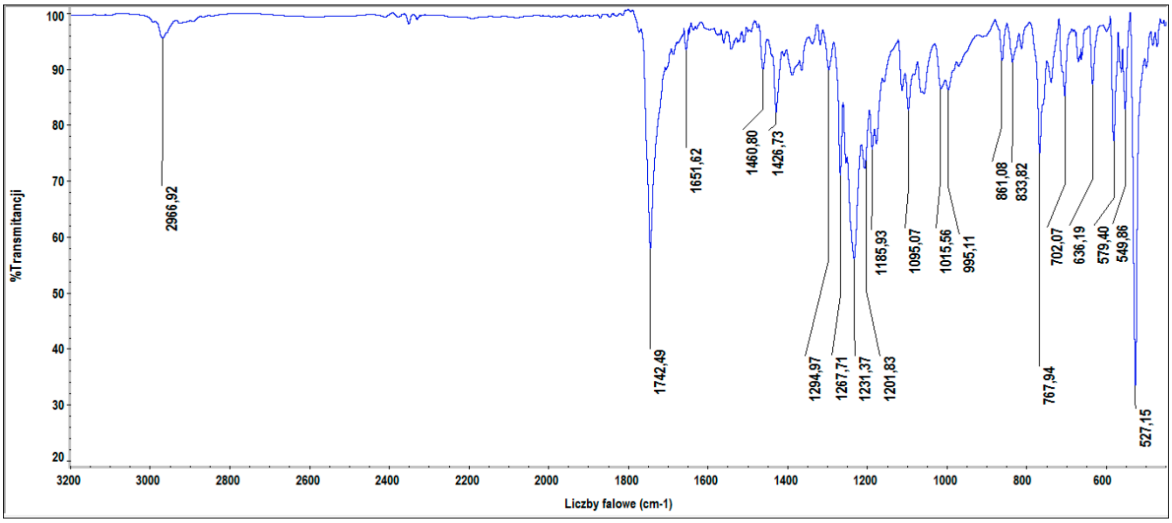

Figure S6. IR spectrum of **[C60]T1** in KBr disk.

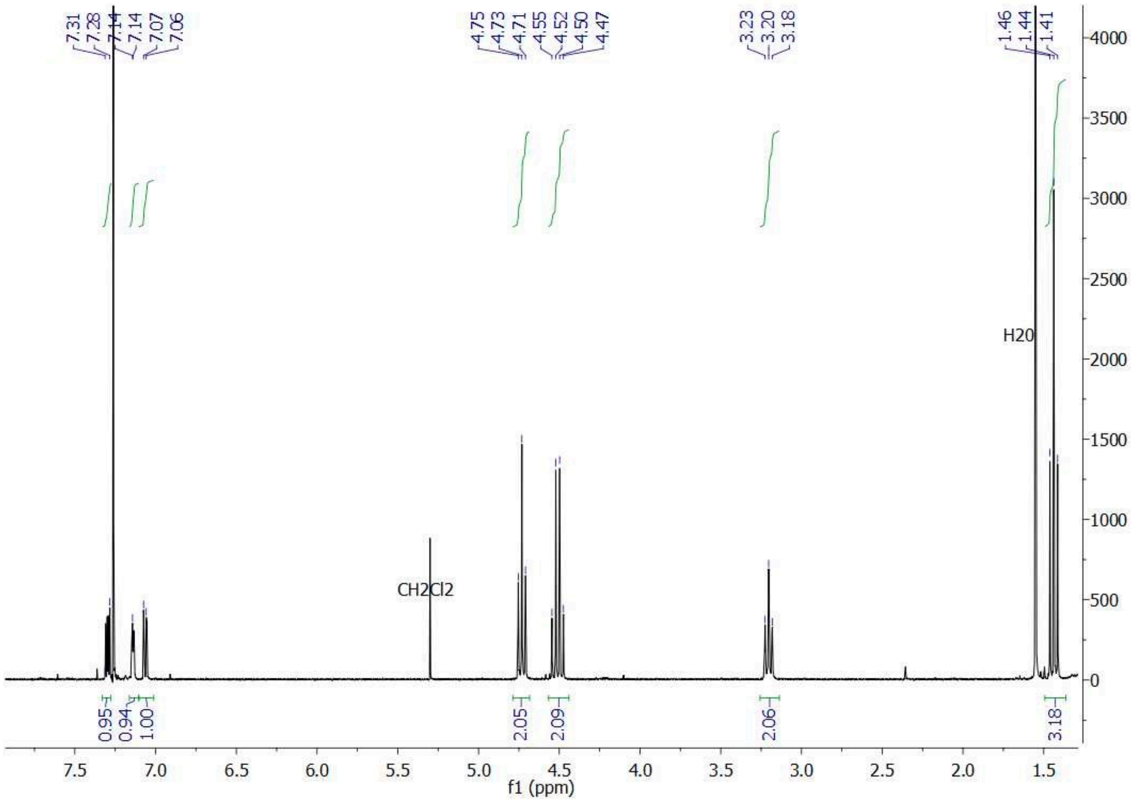

Figure S7. ^{1}H NMR spectrum of **[C60]T1** in $CDCl_3$.

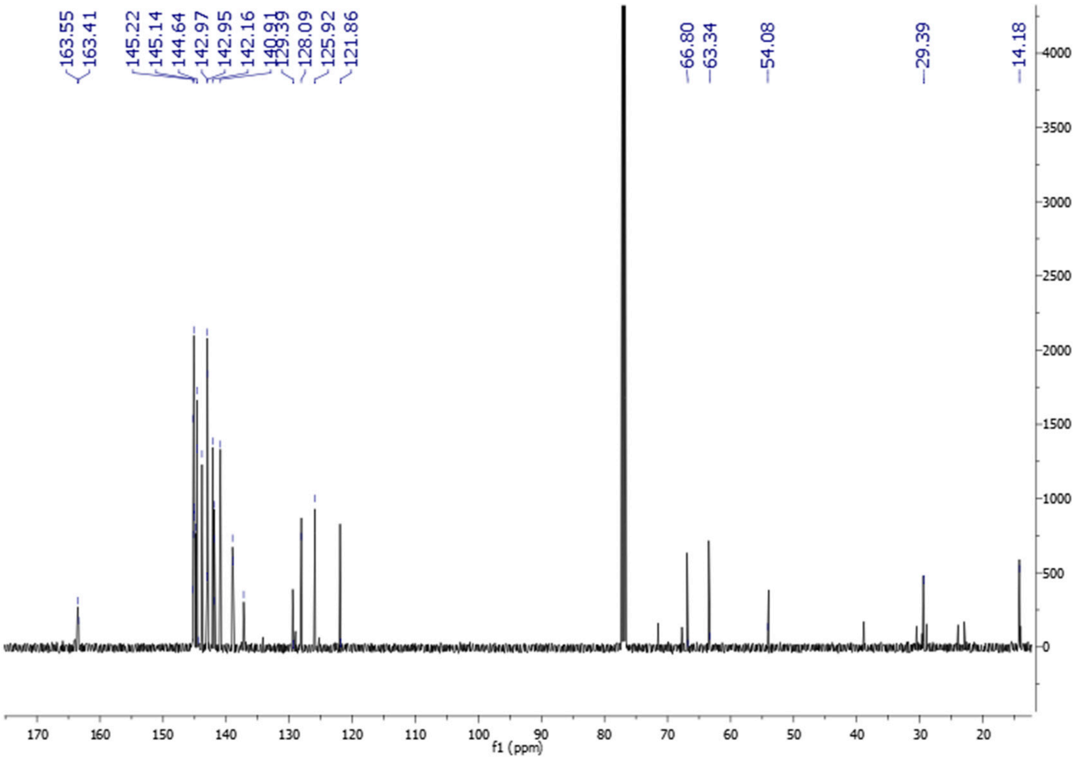

Figure S8. ^{13}C NMR spectrum of **[C60]T1** in $CDCl_3$.

Bis-61-carbonyl o[2-(thiophen-3-ylo)ethylo]-1,2-methano[60]fullerene ([C60]T2)

[C60]T2 was synthesized in two steps using analogous procedure as the one for 1-etyloxycarbonyl-[61-(2-(thiophen-3-ylo)ethylo)]-1,2-methano[60]fullerene.

Bis(2-(thiophen-3-yl)ethyl) malonate

Yield: 56%, ESI-MS: 347.0 $[M+Na]^+$, FT-IR (CCl_4) ν_{max}(cm^{-1}) 2961.03, 2932.45, 2902.35, 2868.71, 1757.17, 1740.01, 1457.67, 1411.14, 1383.92, 1328.75, 1267.97, 1248.78, 1216.91, 1179.73, 1147.75, 1068.19, 1015.30, 862.28; ^1H NMR (500 MHz, $CDCl_3$) δ 7.27-6.95 (m, 3H), 4.34 (t, J = 6.9 Hz, 2H), 3.37 (s, 1H), 2.98-2.97 (m, 2H)ppm; ^{13}C NMR (125 MHz, $CDCl_3$) δ166.35, 128.18, 125.69, 121.69, 65.31, 41.57, 29.35ppm.

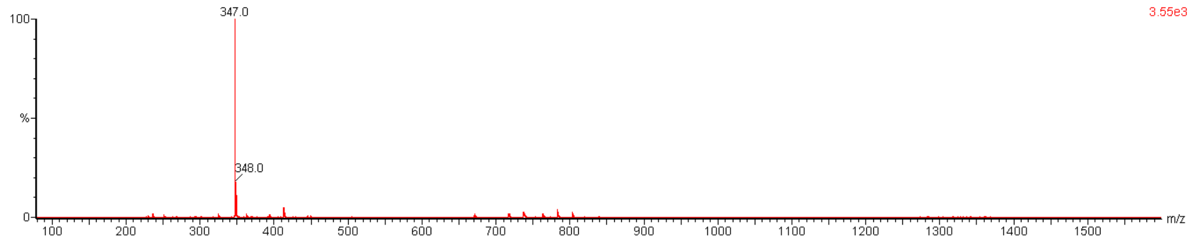

Figure S9. ESI-MS spectrum of bis(2-(thiophen-3-yl)ethyl) malonate as a $[M+Na]^+$ cation.

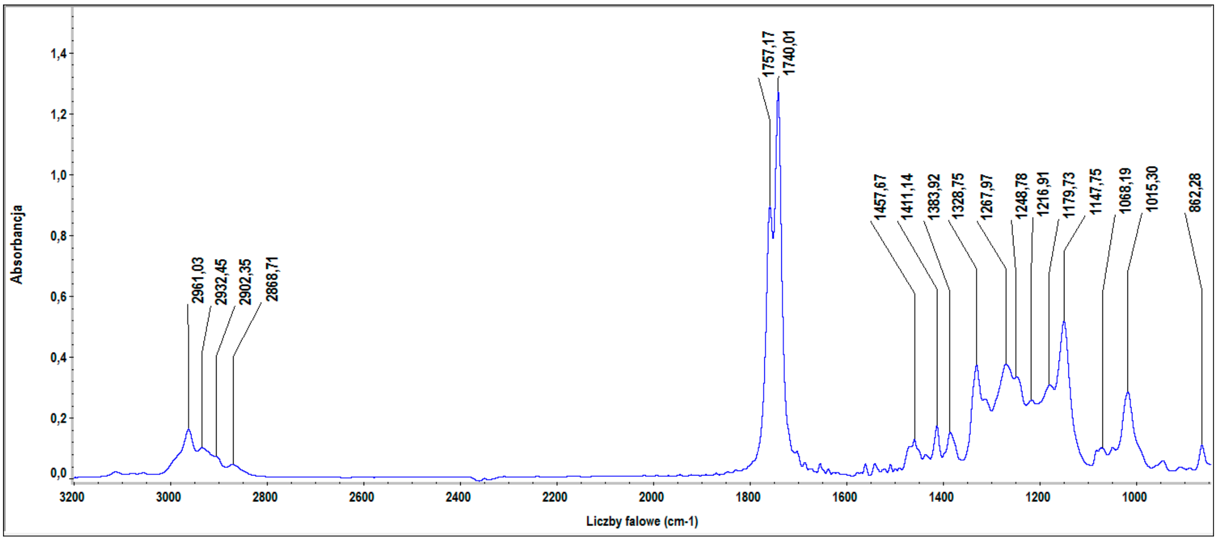

Figure S10. IR spectrum of bis(2-(thiophen-3-yl)ethyl) malonate (CCl_4).

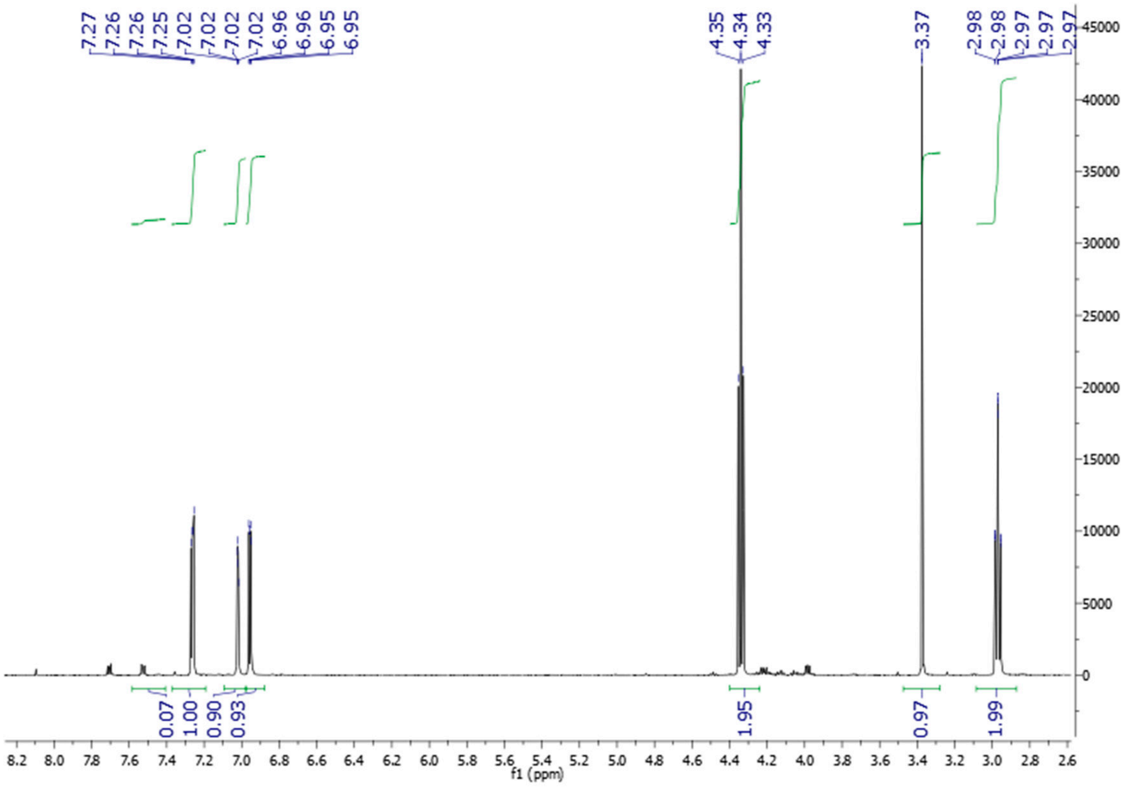

Figure S11. ^{1}H NMR spectrum of bis(2-(thiophen-3-yl)ethyl) malonate in $CDCl_3$.

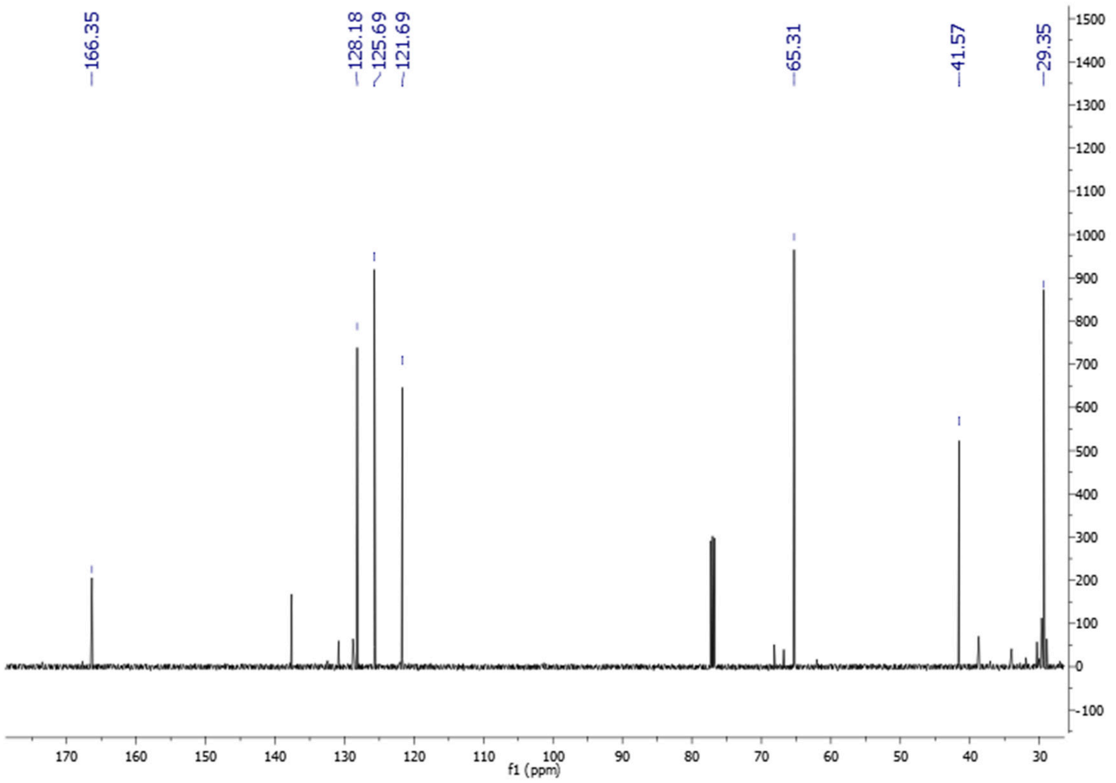

Figure S12. ^{13}C NMR spectrum of bis(2-(thiophen-3-yl)ethyl) malonate in $CDCl_3$.

Bis-61-carbonyl [2-(thiophen-3-ylo)ethylo]-1,2-methano[60]fullerene ([C60]T2)

Yield: 46%, ESI-MS: 1066.2 $[M+Na]^+$), FT-IR (KBr) $\nu_{max}(cm^{-1})$ 2947.59, 2923.00, 1740.24, 1718.17, 1700.34, 1653.29, 1557.17, 1539.29, 1507.99, 1458.81, 1428.50, 1265.78, 1251.47, 1243.75, 1228.25, 1202.93, 1186.52, 1175.26, 1113.39, 1097.11, 780.21, 736.77, 638.42, 578.06, 551.24, 526.89; ^1H NMR (300 MHz, $CDCl_3$) δ 7.29-7.04(m,3H), 4.67 (t, J = 6.7 Hz, 2H), 3.14 (t, J = 6.6Hz, 2H)ppm; ^{13}C NMR (125 MHz, CDCl3) δ 163.47, 145.27, 145.19, 145.13, 145.12, 144.91, 144.70, 144.62, 143.88, 143.03, 143.00, 142.21, 141.92, 140.96, 138.96, 128.15, 125.98, 121.99, 77.28, 77.02, 76.77, 71.43, 68.17, 66.94, 38.74, 30.37, 29.44, 28.93, 23.75, 23.00, 14.07, 10.97, 0.01ppm.

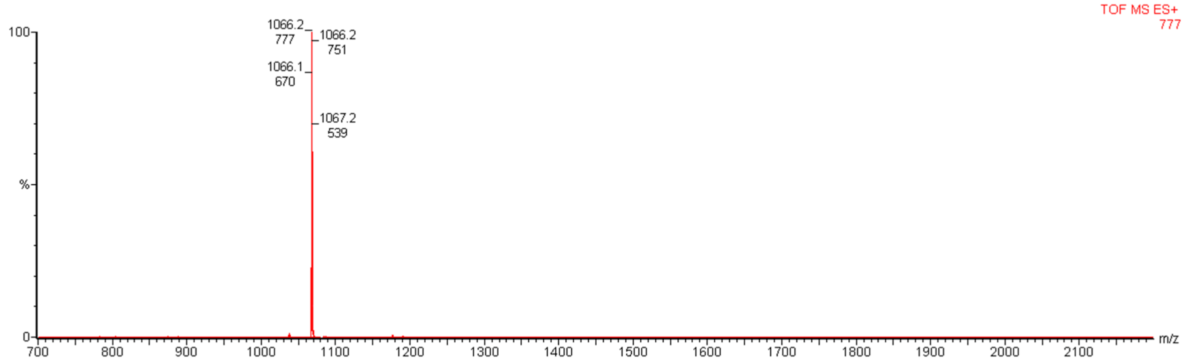

Figure S13. ESI-MS spectrum of **[C60]T2** as $[M+Na]^+$ anion.

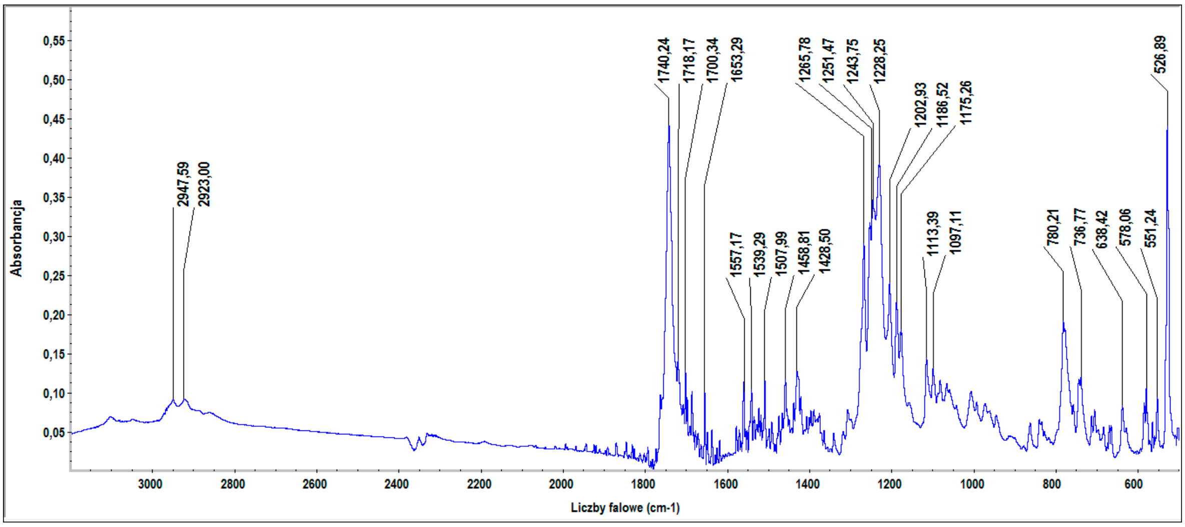

Figure S14. IR spectrum of **[C60]T2** in KBr disk.

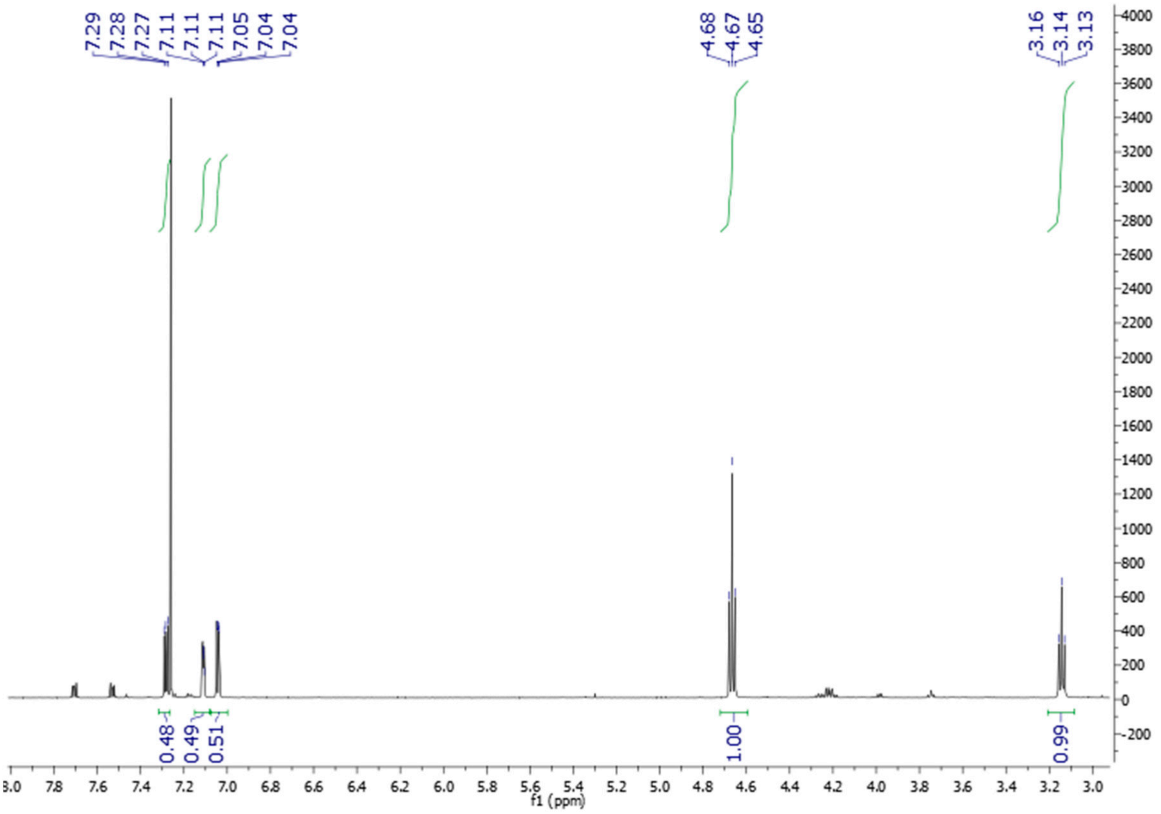

Figure S15. ^{1}H NMR spectrum of **[C60]T2** in $CDCl_3$.

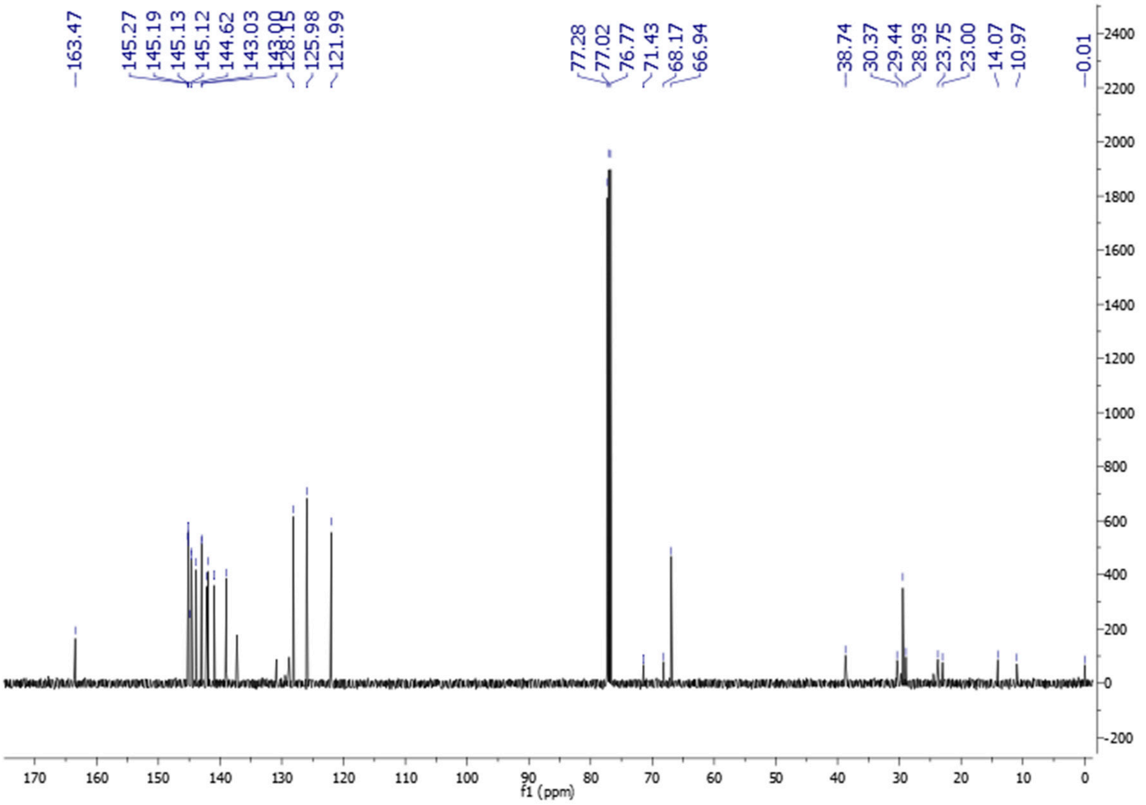

Figure S16. ^{13}C NMR spectrum of **[C60]T2** in $CDCl_3$.

Table S1. Approximate solubilitiy of synthesized fullerene derivatives.

Approximate solubilities were determined with the classical shake-flask method. Briefly, a small amount of compound (2-8mg) was weighed and chlorobenzene was added in 100 μL portions until visually everything looked dissolved. The measurement error is estimated at approx. 25%.

Material	Solubility [mg/ml]
[C60]P1	8
[C60]P2	1.2
[C60]T1	19
[C60]T2	13
PC61BM	25-59.5[1]

2. LESR Studies Supportive Information

1. ESR dark signal of the studied fullerene derivatives and P3HT blends

For ESR measurements samples were prepared with a procedure described in the Methods section.

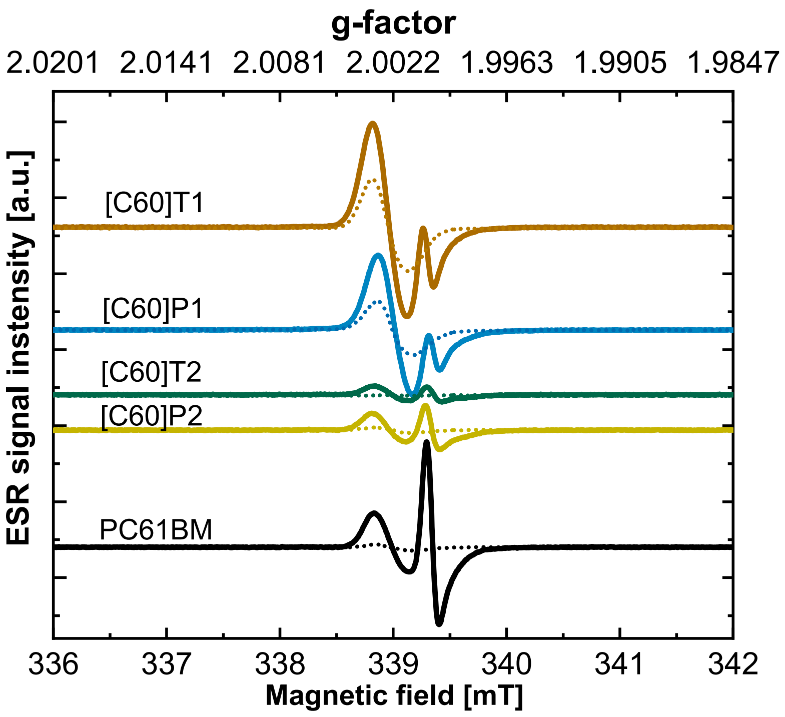

Figure S17. ESR signal of C_{60} fullerene derivatives and P3HT blends at 30 K and 0.0047 mW microwaves power. Dotted lines represent ESR signal in darkness, solid lines – under illumination.

2. g-tensor analysis

Table S2 presents g-tensor components of photo-generated electrons and holes in pure C_{60} fullerene derivatives.

Table S2. g-tensor components for negative (–) and positive (+) polarons located at fullerene sphere and functional group respectively during illumination.

	g_x	g_y	g_z	g_{iso}
PC61BM (–)	2.00065	2.0001	2.0004	1.9998

PC61BM (+)	2.0026	2.0023	2.0012	2.0020
[C60]T1 (–)	2.0005	2.0005	1.9998	2.0003
[C60]T1 (+)	2.0036	2.0024	2.0015	2.0025
[C60]P1(–)	2.0004	2.0005	1.9994	2.0001
[C60]P1 (+)	2.0030	2.0029	2.0025	2.0028
[C60]T2 (–)	2.0005	2.0002	1.9995	2.0000
[C60]T2 (+)	2.0029	2.0022	2.0023	2.0025
[C60]P2 (–)	2.0005	2.0004	1.9996	2.0001
[C60]P2 (+)	2.0034	2.0024	2.002	2.0026
pure P3HT	2.00515	2.0002	2.0019	2.0024

3. LESR signal saturation and line full width at half maximum analysis

The values of the LESR line full width at half maximum (FWHM) for both components of the blends: the P3HT polymer and the studied fullerene derivatives along with the ε parameter values are presented in Table S3. As can be seen, most of the ε parameter values are very close to 0.5 value.

Table S3. The ε parameter values obtained from fitting of LESR signal intensity as a function of microwave power using formula (1), and FWHM values of LESR signals under 0.015 mW, for photo-generated electrons and holes in the fullerene-polymer blends.

Sample	Charge	Temperature [K]	ε value	FWHM [mT]
[C60]T1	(–)	5.4	0.5	0.24
		25.7	0.4	0.18
		59	0.4	0.16
	(+)	5.4	0.7	0.37
		25.7	0.7	0.28
		59	0.4	0.25
[C60]P1	(–)	5.4	0.4	0.17
		25.7	0.5	0.21
		59	0.5	0.16
	(+)	5.4	0.4	0.39
		25.7	0.4	0.22
		59	0.4	0.22
[C60]T2	(–)	5.4	0.5	0.33
		25.7	0.4	0.24
		59	0.5	0.25
	(+)	5.4	0.6	0.33
		25.7	0.5	0.17
		59	0.6	0.19
[C60]P2	(–)	5.4	0.7	0.20
		25.7	0.5	0.26
		59	0.5	0.28
	(+)	5.4	0.5	0.36
		25.7	0.5	0.16
		59	0.5	0.16
PC61BM	(–)	5.4	0.5	0.30
		25.7	0.5	0.16
		59	0.5	0.17
	(+)	5.4	0.4	0.16
		25.7	0.5	0.14
		59	0.5	0.14

3. TRPL Studies

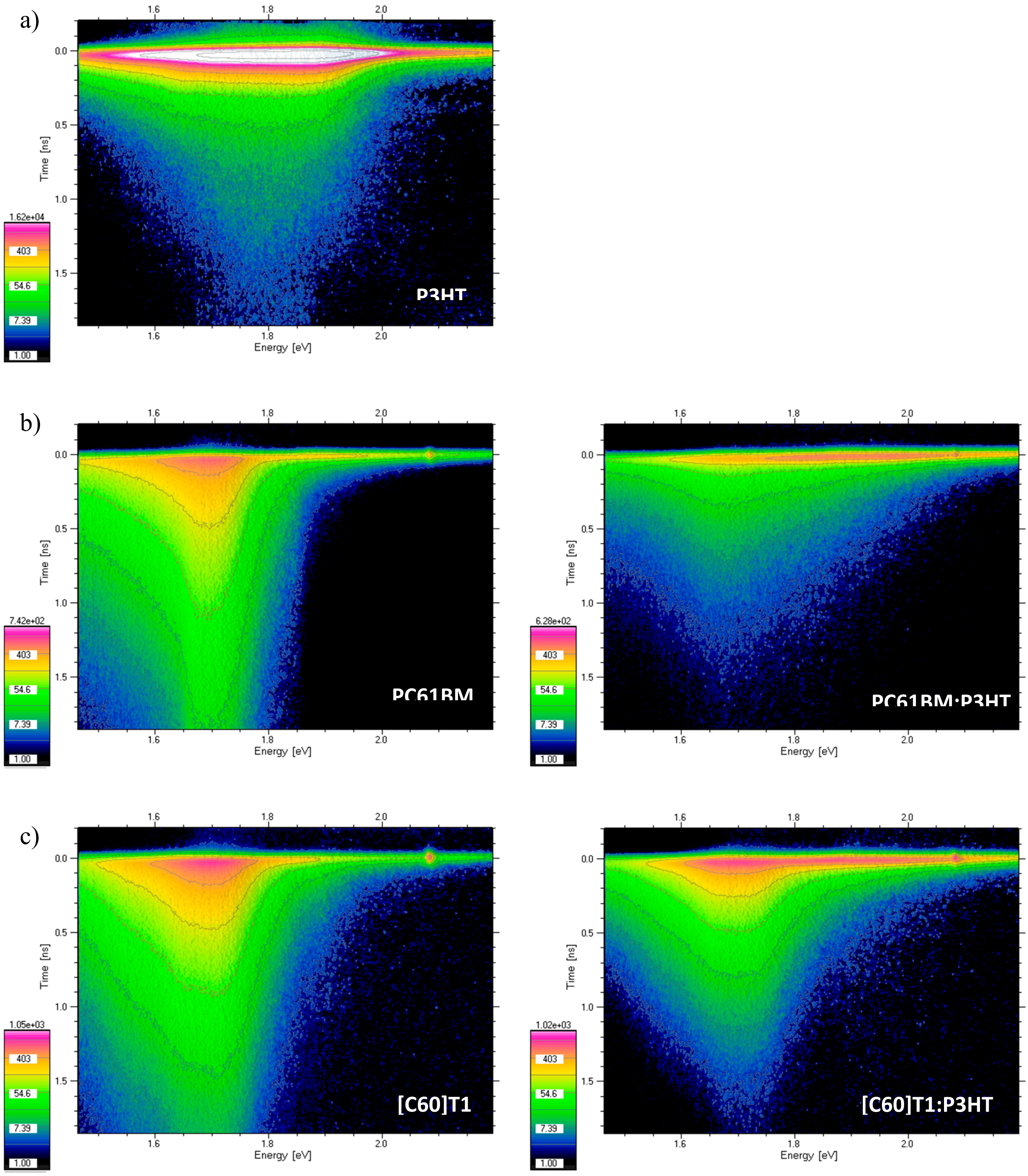

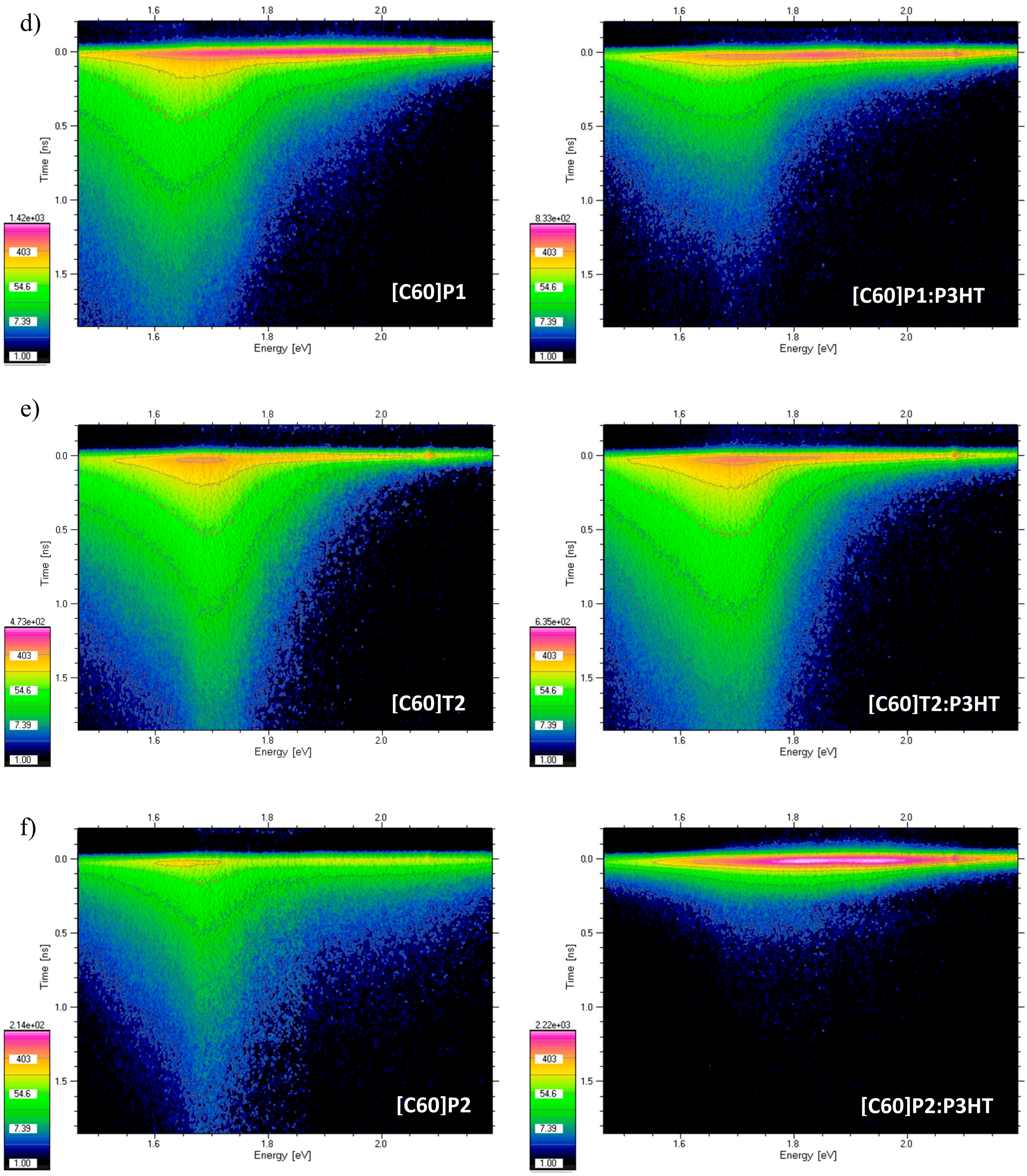

Figure S18 TRPL spectra of a) P3HT and b – f) all studied C_{60} fullerene derivatives and their blend with P3HT. It is visible that all blends attenuate PL compared to pure C_{60} fullerene derivatives

Table S4. Exciton decay time (τ_{blend}), radiative recombination time (τ_{pure} and escape rate calculated from TRPL spectra presented in Figure 9.

	τ_{blend} *[ns]*	τ_{pure} *[ns]*	γ_E *[ns $^{-1}$]*
[C60]P1	0.06	0.39	14.1
[C60]P1:P3HT	0.05	0.36	17.2
[C60]T1	0.12	0.41	5.9
[C60]T1:P3HT	0.09	0.30	7.8
[C60]T2	0.08	0.39	9.9
[C60]T2:P3HT	0.08	0.39	9.9
PC61BM	0.10	0.45	7.8
PC61BM:P3HT	0.04	0.40	22.5

4. DFT Calculations

Table S5. HOMO (also HOMO-1 and HOMO-2 for selected compounds), LUMO energies, and HOMO – LUMO energy differences of the studied fullerene derivatives obtained by DFT calculations. The energy distance between LUMO and the HOMO localized at the fullerene cage for all studied fullerene derivatives is bolded. The calculations are done using the triple-zeta basis sets, i.e. 6-311G (d, p).

**Material**	**PC61BM**	**[C60]T1**	**[C60]P1**	
**LUMO [eV]**	−3.47	−3.54	−3.52	
**HOMO [eV]**	−6.06	−6.08	−5.76	−6.06
**LUMO – HOMO / HOMO-1[eV]**	**2.59**	**2.54**	2.24	**2.54**

**Material**	**[C60]T2**	**[C60]P2**		
**LUMO [eV]**	**−3.57**	−3.49		
**HOMO [eV]**	−6.12	−5.75	−5.76	−6.03
**LUMO – HOMO / HOMO-1 / HOMO-2 [eV]**	**2.55**	2.26	2.27	**2.54**

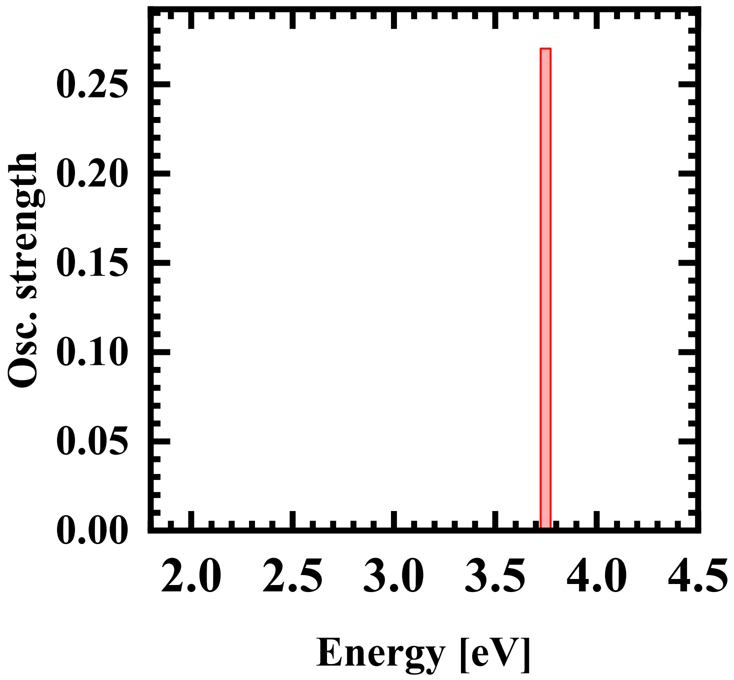

Figure S19. Spectrum of pyrene calculated by DFT. The absorption line at 3.75 eV can be related to the calculated absorption lines of fullerenes [C60]P1 and [C60]P2 at 3.6 eV [Figure 4] and the measured absorption peaks at 3.4 eV ([C60]P2) and 3.5 eV ([C60]P1) [Figure. 3].

5. Solar cell measurements

Current-voltage *J-V* characteristics of solar cells were measured by means of Keithley 2450 Source Meter with Kickstart PC software and using Ossila 8-pixel test board. As a source of illumination a Newport VeraSol-2 LED Class AAA Solar Simulator of 1000 W/m^2 power output and AM1.5G spectrum was applied.

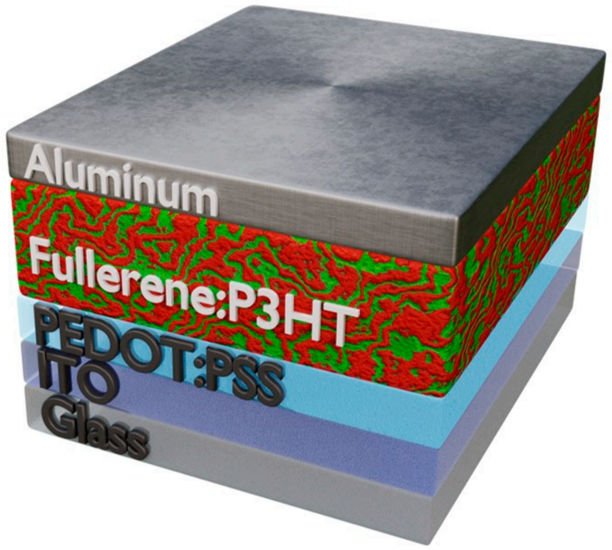

Figure S20. Manufactured organic solar cell scheme.

Polymer-fullerene solar cells were constructed in the glass/ITO/PEDOT:PSS/P3HT:(fullerene derivative)/Al configuration, shown in Figure S20. Glass substrates coated with ITO were cleaned in UV ozone cleaner and then 40 nm-thick PEDOT:PSS layer was dynamically spin-coated on them and annealed at 150 °C for 5 minutes to remove some

residual water molecules. The active layer solutions were prepared in chlorobenzene with a 25 mg/ml blend concentration. The molar ratio of P3HT to the respective fullerene derivative was 2:3. The solutions were heated at 60 °C and stirred for 24 h. After cooling down, they were filtered with a PTFE 0.45 μm syringe filter to remove undissolved excess of fullerene derivative and then deposited on substrates with PEDOT:PSS layer by dynamic spin coating in an argon atmosphere. The active layer thickness was about 90 nm. Finally, after drying the active layer, aluminum-indium cathode was applied using thermal evaporation.

J-V characteristics of illuminated cells are shown in Figure S21, whereas their electrical parameters are presented in Table S5. Among new fullerenes derivatives better were the cells with fullerenes functionalized with one aromatic substituent, i.e. [C60]P1 and [C60]T1. They were characterized by J_{SC} of 1.4 mA/cm^2 and 0.42 mA/cm^2, and their V_{OC} was 0.353 V, and 0.158 V, respectively. These values were still substantially lower than for the reference cell with PC61BM: J_{SC} of 7.4 mA/cm^2 and V_{OC} of 0.555 V. *PCE* of the discussed cells was 0.17% and 0.013%, in comparison with 2.2% of the reference cell. The cells with fullerene derivatives functionalized with two aromatic substituents, [C60]T2 and [C60]P2, underperformed heavily, reaching only 0.001% and 0.006% efficiency.

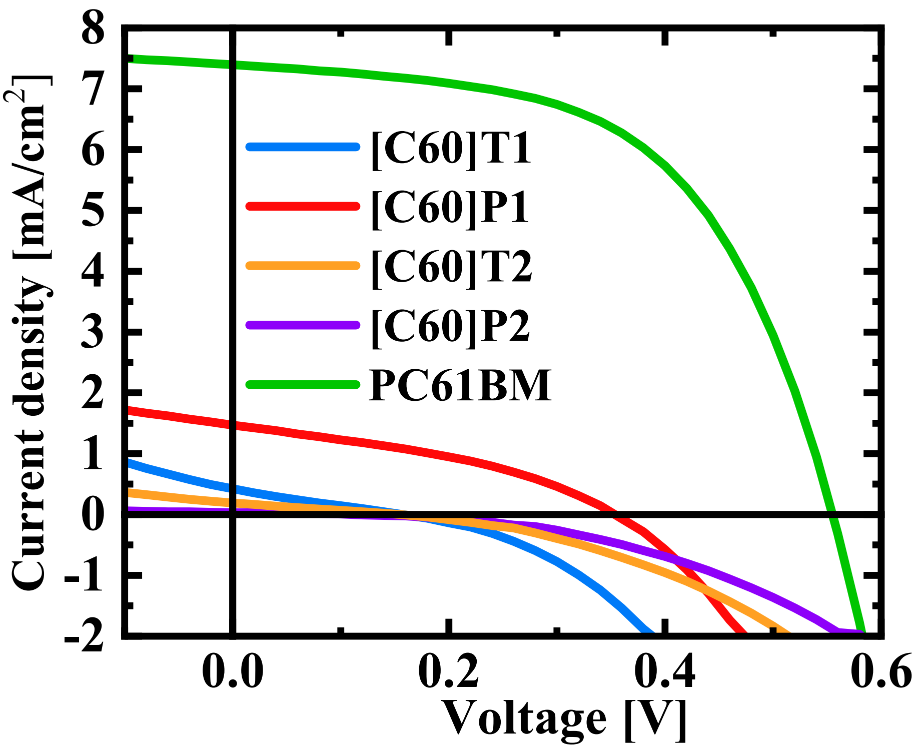

Figure S21. I-V curves of studied solar cells with different active layers.

Table S6. Electrical parameters of manufactured solar cells.

Sample	V_{OC} [V]	J_{SC} [mA/cm^2]	*FF* [%]	*PCE* [%]
**[C60]P1**	0.353	1.47	37	0.17
**[C60]T1**	0.158	0.42	23	0.013
**[C60]P2**	0.111	0.03	29	0.001
**[C60]T2**	0.155	0.19	25	0.006
**PC61BM**	0.555	7.40	56	2.2
